# Physiology and morphology of clonal Atlantic salmon—influence of incubation temperature, ploidy, and zygosity

**DOI:** 10.1007/s10695-025-01533-8

**Published:** 2025-07-17

**Authors:** Malthe Hvas, Fletcher Warren-Myers, Ida B. Johansen, Per Gunnar Fjelldal, Tom Johnny Hansen

**Affiliations:** 1 Animal Welfare Research Group, Institute of Marine Research, Matre, Norway; 2 Sustainable Aquaculture Laboratory – Temperate and Tropical (SALTT), Deakin University, Geelong, Australia; 3 Department of Preclinical Sciences and Pathology, Faculty of Veterinary Medicine, Norwegian University of Life Sciences, Oslo, Norway; 4 Reproduction and Developmental Biology, Institute of Marine Research, Matre, Norway

**Keywords:** Bimodal size distribution, Isogenic fish, Phenotypic variation, Respirometry, Vaterite in otoliths, Ventricle roundness

## Abstract

Isogenic (clonal) fish lines are useful experimental models to study effects of environment versus genetics on phenotypic traits, as they can be maintained for generations without change, providing advantages over outbred groups prone to generational change and higher variation. Here we performed experiments on isogenic Atlantic salmon groups that were either heterozygous diploid, homozygous diploid, triploid, or heterozygous diploid incubated at 4 °C instead of 8 °C. We measured metabolic rates, stress response, and hypoxia tolerance to assess whole-animal performance traits. Then we measured the morphology of hearts and otoliths since both are known to be influenced by environmental history. Isogenic, ploidy, and zygosity statuses were confirmed from microsatellite markers. Embryonic development is affected by temperature, hence the 4 °C incubation group was tested 9 months later when it had reached an equivalent size as the other groups. Curiously, a bimodal size distribution emerged in this group. Physiological traits were similar between groups apart from higher standard metabolic rates in the 4 °C incubated fish. Each group had distinct heart morphologies where fish with a slower growth history resembled wild-phenotypes while homozygous fish had the most deviating hearts. Proportions of vaterite deposition in otoliths showed high individual variation and did not differ between groups. Lower coefficients of variation within groups were found when compared to outbred fish, but this was not consistent for all traits assessed. As such, substantial phenotypic variation in physiology and morphology was still observed in isogenic Atlantic salmon, which can be ascribed to random environmental factors.

## Introduction

To what extent phenotypic traits are influenced by the underlying genetics relative to environmental factors is of fundamental interest in all areas of biological research. In experimental biology this also imposes an inherent challenge as high amounts of individual variation can make it difficult to interpret results, particularly when using random and variable genetic material from different generations and locations (Festing 1976; 1999; Brown et al. 2011). Substantial individual variation is also typically expected, meaning that a high number of replicate animals is required in experimental designs to ensure statistically robust data.

An increased emphasis on animal ethics and welfare in recent years has resulted in stricter regulations for how and when animals are allowed to be used in scientific research. Here, the 3Rs, replacement, reduction, and refinement, is a widespread framework when aiming to improve animal welfare and to reduce the number of animals used in experiments (Hubrecht and Carter 2019). Reduction in animals can be achieved by minimizing the expected individual variation when using less variable genetic material (Bongers et al. 1998). To completely control for the genetic component in the phenotypic outcome of individuals, isogenic (clonal) animals can be used (Komen and Thorgaard 2007; Franěk et al. 2020). Owing to being genetically identical, any phenotypic variation will then be a product of environmental effects. This allows for standardizing experimental animals, which provide an advantage over outbred groups that are subject to generational changes and greater interindividual variation (Festing 1999; Hansen et al. 2020).

In species of fish, the protocols for making isogenic groups require two generations, starting with making homozygous fish (identical alleles on both sets of chromosomes) and these can be either double haploid gynogenetic or androgenetic, depending on which parent gametes are inactivated in the first embryonic mitosis following fertilization (reviewed by Komen and Thorgaard 2007 and Franěk et al. 2020). Since homozygosity implies being completely inbred, progeny may often not be viable owing to harmful alleles. However, fish have high fecundities, and some homozygous individuals will typically survive and be able to mature and reproduce (Müller-Belecke and Hörstgen-Schwark 2000). The progeny will then obtain identical genetic inheritances from a homozygous parent. By crossing two homozygous parents, all individuals in the next generation will become heterozygous and isogenic. Additionally, isogenic homozygous groups can be made by fertilizing eggs from a homozygous female with UV irradiated sperm, followed by blocking of the second meiotic division. Once a homozygous clonal fish line of interest has been established, it can be maintained for generations without genetic change. Methods for making isogenic fish groups have been established for several species over recent decades and include zebrafish (*Danio rerio*), Nile tilapia (*Oreochromis niloticus*), common carp (*Cyprinus carpio*), rainbow trout (*Oncorhynchus mykiss*), and more recently Atlantic salmon (*Salmo salar*) (Streisinger et al. 1981; Sarder et al. 1999; Komen et al. 1991; Quillet 1994; Hansen et al. 2020).

Atlantic salmon is an economically important aquaculture species that has become highly domesticated through selective breeding (Teletchea et al., 2014; Glover et al. 2017a). Meanwhile it is also a popular species for recreational fishing where declining wild populations are subject to substantial on-going conservation research and management (Dadswell et al. 2021; Johnsen et al. 2020). The genetics and phenotypic outcome of both domesticated and wild Atlantic salmon have therefore been extensively studied (Lien et al. 2016; Glover et al. 2017a).

Owing to a wide interest in Atlantic salmon biology, it has been argued that standardized isogenic lines could become a potentially important tool for numerous fundamental and applied research purposes in this species (Grimholt et al. 2009; Hansen et al. 2020). So far, few experimental studies have been published on isogenic Atlantic salmon. One noteworthy example compared sea lice infestation levels between isogenic and outbred Atlantic salmon groups and found that while parasite load was highly variable between individuals, it was unrelated to the underlying genetics of the fish (Glover et al. 2017b). This provides an interesting example of how random non-genetic factors in experimental conditions can be a primary cause for individual variation in a trait known to be strongly related to long-term survival chances (Bui et al. 2024). However, additional work is needed where more phenotypic traits are quantified to better demonstrate the usefulness of such isogenic experimental models.

In the present study, we aimed to measure various phenotypic traits in heterozygous diploid, homozygous diploid, and heterozygous triploid (three instead of two sets of chromosomes) group variants all originating from a common isogenic female. Additionally, the isogenic and heterozygous diploid groups were incubated both at 4 °C (representative river temperature in winter) and 8 °C (standard practice in aquaculture) to investigate effects of different embryonic development rates.

First, to assess whole-animal physiological performance, we measured metabolic rates at rest, during acute stress and subsequent recovery, as well as the response and tolerance to progressive environmental hypoxia. Such metrics were considered relevant as energetics and aerobic constraints are traits widely used for inferring robustness and adaptations of fish in various contexts (Fry 1971; Claireaux and Lefrancois 2007; Clark et al. 2013). Furthermore, the metabolic phenotype of fish is well known to both be strongly influenced by environmental factors and to vary substantially between individuals (Metcalfe et al. 2016).

Then, to investigate morphological traits of interest we focused on hearts and otoliths that both show high phenotypic variation with potential implication for fitness. For instance, cultured salmonids have differently shaped ventricles than wild fish (Poppe et al. 2003), and heart morphology of salmonids is also greatly affected by acclimation temperature and growth history (Anttila et al. 2014a; Frisk et al. 2020; Vindas et al. 2024). Furthermore, certain morphological traits such as less round and more pyramid-shaped ventricles are associated with better health and improved physiological performance (Claireaux et al. 2005; Anttila et al. 2014b; Frisk et al. 2020). Egg incubation temperatures above 10 °C have been linked to development of severe cardiac deformities such as missing septum transversum (Nathanailides et al. 1995; Ørnsrud et al. 2004; Takle et al. 2005). However, the impact of currently used egg incubation temperatures on more subtle aspects of geometric heart shape (e.g., rounding of ventricle) during embryological development remains unknown.

Otoliths are key components of the vestibular system in fish, but improper development thought to occur at early-life stages can lead to deformities which impair hearing and swimming balance (Tomas and Geffen 2003; Oxman et al. 2007; Brown et al. 2013). These deformities consist of a change in the calcium carbonite polymorphs in the otolith structure where vaterite replaces aragonite (Carlström, 1963; Popper and Lu 2000). Domesticated Atlantic salmon with abnormal vaterite formation in otoliths is more common than in wild counterparts (Reimer et al. 2016). In addition, these deformities are more prevalent in individuals reared in rapid growth environments (Reimer et al. 2017).

We hypothesized that several differences in physiological and morphological traits would be found between the isogenic groups owing to the respective influence of zygosity, ploidy and thermal growth history. For instance, the lower incubation temperature was expected to provide morphological phenotypes more similar to wild-types with less vaterite deposits in otoliths and more pyramid shaped ventricles that in addition should improve aerobic capacities. Meanwhile, homozygous fish were expected to be more deformed and to perform the worst in the physiological tests owing to the disadvantage of being functionally inbred. Triploids have an extra set of chromosomes, resulting in larger cells with lower surface to volume ratios relative to diploids, and this could impose exchange rate limitations across cells leading to reduced whole-animal physiological functioning and thereby a lower tolerance to stress and hypoxia along with elevated basal maintenance costs from having 50% larger genomes (Hansen et al. 2015; Riseth et al. 2020). Finally, we hypothesized that less individual variation would be observed within isogenic groups when compared to previous measurements in outbred Atlantic salmon, which would demonstrate the general advantage of isogenic animal models in experimental biology.

## Methods

### Production of clonal Atlantic salmon and animal husbandry

The method used to make isogenic Atlantic salmon were previously described in Hansen et al. (2020). On 16. November 2020, eggs from a doubled haploid (i.e., homozygous) Atlantic salmon female were divided into 4 groups of approximately 2000 eggs. To make the homozygous isogenic line, one egg group was fertilized with diluted and UV irradiated sperm and pressurized for 5 min at 655 bar 300 minC postfertilization (second meiotic division).

To make a heterozygous isogenic line, two egg groups were fertilized with frozen milt from a doubled haploid male (see Fjelldal et al. 2020 for details). After fertilization, these two groups were incubated either at 4 °C or 8 °C.

To make a heterozygous triploid isogenic line, one egg group was fertilized with frozen milt from a doubled haploid male and pressurized for 5 min at 655 bar 300 minC (second meiotic division) post-fertilization.

Each group was incubated in a single tray and maintained at their respective incubation temperature (4 °C or 8 °C) prior to first feeding. When ready to being fed at ≈800-degree days (day × temperature), each group were moved to single square, gray, covered, fiberglass tanks (1 × 1 × 0.43 m) supplied with freshwater of 13 °C. When the groups were 10 months old, corresponding to 3338-degree days for the 8 °C incubated groups and 2112-degree days for the 4 °C incubated group,, the fish were again moved to larger tanks (1.5 × 1.5 × 0.7 m). Here, they were reared at ambient seasonal freshwater temperatures under continuous light while being fed continuously using automatic feeders and commercial feeds which were increased in pellet size as the fish grew until the termination of the study. The various holding tanks used were all supplied with filtered, and UV treated water from a flow-through system, ensuring high water qualities at all time. For temperature control, adequate mixing of ambient, cooled, and heated water reservoirs was used.

**Fig. 1:**
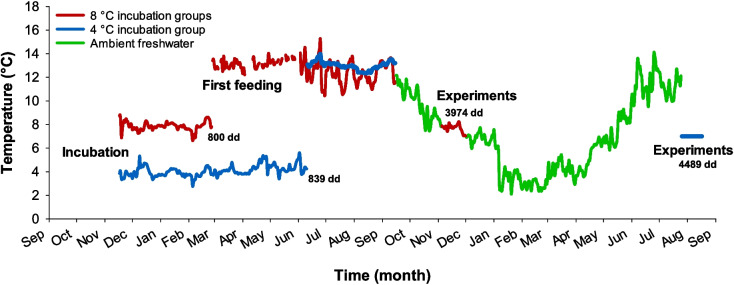
Recorded thermal environment of the isogenic Atlantic salmon groups from incubation to experimental trials. The period with ambient freshwater followed seasonal temperatures. Water was maintained at 7 °C during respirometry trials, and for the slow growing 4 °C incubation group, fish were acclimated at 7 °C for a minimum of 2 weeks before testing. Numbers indicate degree days (dd) at first-feeding, and at the mid-point of experimental trials for the 8 °C and 4 °C incubation groups

Experimental trials in the groups incubated at 8 °C were performed in November and December 2021 when the fish had reached a size of ≈150 g. Here, the test temperature used was 7 °C which represented the ambient seasonal freshwater temperature at this time. The fish incubated at 4 °C developed much slower and experimental trials were therefore first performed 9 months later in August 2022 to allow them to reach an equivalent size as the other groups. Additionally, they were acclimated to 7 °C for a minimum of 2 weeks to provide a comparable recent thermal environment as in the other groups. The thermal history from incubation to experimental trials of the isogenic Atlantic salmon groups are shown in Fig. 1.

The fish from the low incubation temperature group had developed a bimodal distribution in sizes. Around one-third of the fish were much smaller than the others. These fish were not tested experimentally, but 16 fish were sampled for morphological analyses.

This study was approved by the Norwegian Food Safety Authority under permit number 28553 and 29,835 for appropriate use of animals in scientific research.

### Respirometry setup

Oxygen uptake rates (ṀO_2_) as a proxy for aerobic metabolic rates in the isogenic Atlantic salmon groups were measured with an intermittent flow respirometry system (Loligo Systems, Denmark). This system consisted of 4 cylindrical acrylic chambers that all were 30 cm long with an internal diameter of 8 cm, allowing 4 appropriately sized individual fish to be tested in parallel. These chambers were connected to a circulation pump and a flow-through oxygen sensor cell via gas-tight plastic tubes which formed an internal loop to ensure a homogenous aquatic environment. The volume of this closed system was measured to be 1.584 l. To allow for intermittent flushing, the chambers were also connected to a flush pump (5 l min^−1^) via an open loop. The 4 respirometry chambers together with tubes, pumps, and oxygen sensor were each submerged in separate rectangular tanks with an approximate water volume of 140 l. To maintain a constant water temperature and to reduce waste accumulation as well as to mitigate bacterial proliferation during the experiments, a constant water flow was running in and out of each tank. The source of water was of the same quality as provided in the fish holding tanks at the time of the experimental trials (7 °C, aerated freshwater). Each oxygen sensor was carefully calibrated in accordance with the manufacturer’s instructions before starting the experiment and connected with an optic fiber cable to a computer running the AutoResp software (Loligo Systems, Denmark) together with the flush pumps. The computer software was then used to control periodic flushing automatically and to log oxygen concentrations in each respirometry chamber at 1 Hz. This entire setup was installed in its own secluded room to ensure that potential unwanted disturbances were avoided.

### Experimental protocols

Respirometry trials were performed using diploid, triploid, and homozygous isogenic Atlantic salmon incubated at 8 °C, and on diploid isogenic Atlantic salmon incubated at 4 °C. Sixteen fish were tested individually from each of these 4 groups. Furthermore, in the low incubation temperature treatment, the fish had an obvious bimodal size distribution. As such, 16 clearly undersized fish were sampled to document size differences, but these were not tested with respirometry.

To prepare for a respirometry trial, 4 random fish were moved to an empty tank of similar properties as the regular holding tanks in the afternoon to facilitate fasting overnight. This was done to reduce the confounding metabolic effects of specific dynamic action. The next day fish were individually netted and transferred in a bucket to the secluded respirometry room. They were then moved into their designated chamber that was sealed off as quickly as possible whereafter ṀO_2_ measurements were immediately started. Here, the first measurement period sought to capture the combined response following acute handling stress and initial confinement stress in a novel space-restricted environment. In the case of Atlantic salmon this has been shown to provide a higher ṀO_2_ when compared to the conventional procedure of chasing the fish to exhaustion beforehand when seeking to obtain a measurement of the maximum metabolic rate in static respirometers (Hvas and Oppedal 2019).

Once inside the chamber, the measurement protocol began and consisted of a 3-min closed period followed by 1.5 min of flushing to reestablish oxygen levels and a 0.5-min wait period to stabilize the water flow prior to the next closed period. This cycle was repeated automatically overnight for 24 h while fish were kept in dim lit conditions to make them calmer.

The next day after 24 h had passed, the response to progressive hypoxia was measured by omitting the flush and wait periods in the measurement cycle. This gradually reduced oxygen concentrations within the chamber. Once a critical level was reached where the ṀO_2_ became dependent on PO_2_, as identified from a linear relationship between ṀO_2_ and PO_2_ in the AutoResp software, the chamber was flushed again to conclude the trial. This was done prior to loss of equilibrium of the fish. Then the fish was removed from its chamber and euthanized in a lethal dose of anesthetics (300 mg l^−1^ tricaine mesylate, Finquel Vet.), and the empty respirometer was resealed to quantify background respiration rates over 3–4 measurement cycles.

Thereafter the weight and fork length of the fish were measured. The adipose fin was cut off and preserved in 70% ethanol solution for later microsatellite DNA analyses to confirm clonal, ploidy, and zygosity status. The heart was carefully dissected out and momentarily submerged in a saline solution (137 mM NaCl, 2.7 mM KCl, 10 mM Phosphate buffer) to facilitate blood emptying followed by gently cleaning with a paper towel before being stored in a 70% ethanol solution for later morphological analyses. The remaining carcass of the fish was stored in a freezer at − 18 °C for later dissections of the sagittal otoliths. Finally, to prepare for the next trial, the chambers, pumps, and tubes were disassembled and cleaned in warm soapy water before being reassembled.

### Confirmation of isogenic, zygosity, and ploidy identity

From the adipose fin sampled from each fish microsatellite DNA analyses were performed to confirm isogenic, zygosity and ploidy status. Genotyping DNA extracted from fin-clips was performed in 96-well plates using a commercially available extraction kit (Qiagen DNeasy®96 Blood & Tissue Kit). Each 96-well plate included two blank wells as negative controls. The samples were subject to genotyping with a set of microsatellites that are routinely used in the molecular genetics’ laboratory at the Institute of Marine Research, Norway, for Atlantic salmon genetics projects (e.g., Glover et al. 2012).

The samples were analyzed with 19 microsatellites. These loci were amplified in three multiplexes, using standard protocols for fresh tissues: SSsp3016 (Genbank no. AY372820), SSsp2210, SSspG7, SSsp2201, SSsp1605, SSsp2216 (Patterson et al. 2004), Ssa197, Ssa171, Ssa202 (O’Reilly et al. 1996), SsaD157, SsaD486, SsaD144 (King et al. 2005), Ssa289, Ssa14 (McConnel et al. 1995), SsaF43 (Sanchez et al. 1996), SsaOsl85 (Slettan et al. 1995), MHC I (Grimholt et al. 2002), and MHC II (Stet et al. 2002). Polymerase chain reaction (PCR) products were analyzed on an ABI 3730 Genetic Analyzer and sized by a 500LIZ™ size-standard. The raw data was checked manually twice.

The clonal status could be confirmed by identical number of DNA repetitions in the assessed microsatellite markers between individuals. Heterozygosity or homozygosity were confirmed from the presence or absence of two alleles for the various microsatellites assessed, respectively. Triploidy could be inferred from a higher amplification in those alleles that were doubled (Delaval et al. 2024).

### Heart morphology

Fixed hearts were placed in water, attached to a pushpin at the bottom of a transparent plastic box on a light plate (Slimlite LED,32 × 22.8 cm, Kaiser, Buchen, Germany). Photographs of the ventrodorsal and left lateral views of the heart were captured from above utilizing a Canon Powershot SX540 HS (Canon Inc., Tokyo, Japan) camera for morphometric examination. Reported morphology traits were quantified as described by Engdal et al. (2024).

To briefly summarize, in the ventrodorsal projection the ventricular height to width ratio was quantified by dividing ventricle length by ventricle width, and relative bulbus size was expressed as bulbus width divided by ventricle width. In the same projection, the apex angle was measured by first dividing the ventricular height into four equal segments, starting from the apex, and extending to the bulbus, followed by drawing a horizontal line at the boundary between the first and second segments, starting at the right and ending at the left longitudinal ventricular ridges. The apex angle was then found from the three points where the horizontal line intersected the right longitudinal ventricular ridge, the apex, and the point where the horizontal line intersected the left longitudinal ventricular ridge.

Then, in the left lateral projection, the ventricular bulbus angle was quantified as the angle between the bulbular horizontal axis and the ventricular vertical axis. The ventricular symmetry was quantified by measuring the angle between the ventricular vertical axis and the axis running from the ventriculobulbar groove to the left dorsal ventricular apex. Lastly, the bulbus alignment was quantified as the angle between the lines running from the ventriculobulbar groove to the left dorsal ventricular apex and from the ventriculobulbar groove to the atriobulbar incision.

### Proportion of vaterite in sagittal otoliths

Dissected and cleaned sagittal otoliths from the clonal Atlantic salmon were placed sulcus side up on a black petri dish before being photographed under a dissecting microscope set at × 10 magnification, connected to a desktop computer. Once all otoliths were photographed, the proportions of vaterite and aragonite in each otolith were measured using the polygonal lasso tool in Adobe Photoshop. For each otolith, we outlined and recorded the total area of the opaque aragonite structure (in pixels), and then repeated this process to measure the total area of the whole otolith. From this, we calculated the proportion of aragonite structure in each otolith by dividing the total area of aragonite (in pixels) by the total area of the otolith (in pixels). The proportion of vaterite was then expressed as 1 minus the proportion of aragonite.

### Calculations and data analyses

The ṀO_2_ was calculated in all the closed measurement periods from the decrease over time in dissolved oxygen as:

$$\overset.{\mathrm M}{\mathrm O}_2=\frac{\frac{\bigtriangleup{\mathrm O}_2}{\bigtriangleup\mathrm t}\left({\mathrm V}_{\mathrm{sys}}-{\mathrm V}_{\mathrm b}\right)}{{\mathrm M}_{\mathrm b}}$$

where ΔO_2_/Δ*t* is the rate of the decrease in oxygen, *V*_sys_ is the volume of the closed system, and *V*_b_ and *M*_b_ are the volume and mass of the fish, respectively. The *R*^2^ of the linear regressions used to calculate ṀO_2_ were most often > 0.98. If *R*^2^ fell below 0.9, those data points were omitted from further analyses. In addition, background respiration rates as measured in the empty chambers were subtracted from all data points. Owing to the relatively low temperature and thorough routine cleaning, these values were very small and presumably mostly represented random fluctuations of the oxygen sensor.

The standard metabolic rate was derived from the mean of the 10% lowest ṀO_2_ values obtained in the first 24 h of the protocol prior to the hypoxia test. If outliers were found, defined as ± 2 standard deviations from this mean, they were removed and a new mean was calculated (Clark et al. 2013).

 The maximum metabolic rate was defined as the highest ṀO_2_ measured which coincided with the first measurement period at the onset of the trial where the fish were most stressed. An absolute and a factorial aerobic scope were subsequently calculated as maximum minus standard metabolic rate and maximum divided by standard metabolic rate, respectively.

The critical oxygen tension (*P*_crit_) was derived at the oxygen level where ṀO_2_ decreased below the standard metabolic rate measured in normoxia (Ern et. 2016; Reemeyer and Rees 2019).

The condition factor of each fish was calculated as 100 · weight (g) · (length (cm)^3^) ^−1^ as a standard morphometric measure in fish research (Fulton 1904; Nash and Valencia, 2006).

To provide a standardized expression of data variation in the measured parameters, the coefficient of variation was calculated as (standard deviation/mean) · 100. These values were then compared to similar experimental work on outbred Atlantic salmon to assess whether clonal fish groups showed less individual variation.

Statistical differences between treatment groups in size, physiological, and morphological traits were assessed with a one-way ANOVA followed by a Tukey post hoc test to see which group differed after first having confirmed normality and equal variance of the data with the Shapiro–Wilk and Levene’s mean tests, respectively. To adhere to test assumptions, it was sometimes necessary to perform a log transformation of the data, and if this was not sufficient an ANOVA on ranks was used instead together with Dunn’s post hoc test. To compare the coefficient of variation in size and metabolic rate parameters from the present study on isogenic groups with outbred fish groups from previous studies, a two-way ANOVA was used followed by Tukey post hoc tests after having confirmed normality and equal variance with the Shapiro–Wilk and Levene’s mean tests. Data analyses were performed in SigmaPlot (v. 14.5 Systat Software). *P*-values below 0.05 were considered significant. Data are reported in the text as the mean ± standard error of the mean unless specified otherwise.

## Results

### Isogenic, ploidy, and zygosity status

The 19 microsatellite markers confirmed that all individual fish were indeed isogenic and that they had the correct ploidy and zygosity in accordance with their group (Table 1). As such, all individuals within a group had the same alleles (number of DNA repetitions) for the microsatellite markers, the triploids had three alleles (of which two were identical) per marker, and the homozygous group had identical alleles on both sets of chromosomes.

**Table 1:** Overview of the 19 microsatellite markers used to confirm isogenic, ploidy, and zygosity statuses. As all fish were isogenic, the number of DNA repetitions were identical for all assessed alleles between individuals within groups. Heterozygous diploids and triploids have two and three sets of chromosomes, respectively. Homozygous diploids have identical alleles on both sets of chromosomes. *N* = 16

Marker	Diploid		Triploid			Homozygous		Slow diploid	
SSsp2201	275	299	275	299	299	299	299	275	299
SSsp2210	136	148	136	148	148	148	148	136	148
SSspG7	127	179	127	179	179	179	179	127	179
Ssa202	246	258	246	258	258	258	258	246	258
SsaD144	166	186	166	186	186	186	186	166	186
SsaD157	343	375	343	375	375	375	375	343	375
SexVIC	Male-4	Male-2	Male-4	Male-2	Male-2	Female_b	Female_b	Male-4	Male-2
Sp1605	244	216	244	216	216	216	216	244	216
Sp2216	226	258	226	258	258	258	258	226	258
Ssa14	140	140	140	140	140	140	140	140	140
Ssa171	213	233	213	233	233	233	233	213	233
Ssa289	122	118	122	118	118	118	118	122	118
MHC I	146	142	146	142	142	142	142	146	142
MHC II	260	370	260	370	370	370	370	260	370
SSsp3016	90	130	90	130	130	130	130	90	130
SsOsl85	195	193	195	193	193	193	193	195	193
Ssa197	216	184	216	184	184	184	184	216	184
SsaD486	172	172	172	172	172	172	172	172	172
SsaF43	121	115	121	115	115	115	115	121	115

### Growth history and size parameters

Early survival rates up to the point of first feeding in the isogenic groups were similar between diploids and triploids being 92.4% and 92.2%, respectively. Meanwhile, the homozygous group had a lower early survival rate of 82.3%. For the latter part of the fish husbandry period, mortalities were negligible in all groups. Prior to the respirometry trials, it was observed that the homozygous group had many individuals with notable spinal deformities, and those individuals were avoided for use in the experiments. We did not quantify the frequency of deformed homozygous fish in this trial.

The fish groups incubated at 8 °C were developing faster and were hatched and first fed 2 and 3.5 months earlier, respectively, than the group incubated at 4 °C. However, expressed as degree day hatching and first feeding was started at similar times at ≈500 and ≈800 degree days, respectively, in the different groups. Altogether the fish incubated at 4 °C required an additional 9 months of growth to reach similar sizes as the faster growing groups had attained at the time of their experimental trials in November and December 2021. These additional 9 months involved a winter of cold ambient freshwater with little growth followed by higher growth as temperatures increased in spring and summer prior to the experimental trials in August 2022 (Fig. 1). The midpoint of the experimental trials with the 8 °C incubation groups occurred after 3974-degree days while the midpoint of the trials for the 4 °C incubation group occurred after 4489-degree days. This corresponded to 13% more degree days for the 4 °C incubation group at the time of testing.

**Fig. 2:**
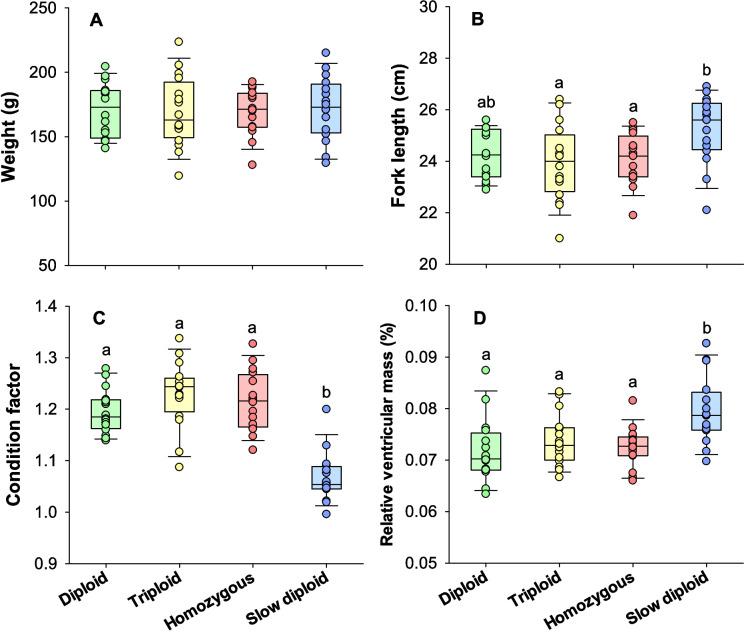
Size parameters of isogenic Atlantic salmon groups at the time of experimentation. Panels show boxplots together with individual datapoints (*N* = 16). Statistical differences between groups are indicated with different letters (one-way ANOVA with Tukey test, *P* < 0.05)

The size parameters at the time of the respirometry trials are summarized in Fig. 2. The weight was similar between all the groups (one-way ANOVA, *DF* = 63, *P* = 0.986) (Fig. 2A). The fork length was significantly longer in the slow diploid group than the triploid and homozygous groups (one-way ANOVA and Tukey test, *DF* = 63, *P* = 0.014 and 0.037, respectively) (Fig. 2B). Additionally, the slow diploids had a lower condition factor, and a higher relative ventricular mass compared to the three other groups (one-way ANOVA, *DF* = 63, *P* < 0.001 in both cases) (Fig. 2C and D). Absolute ventricle mass was similar across groups (one-way ANOVA, *DF* = 63, *P* = 0.212) (not shown).

**Fig. 3:**
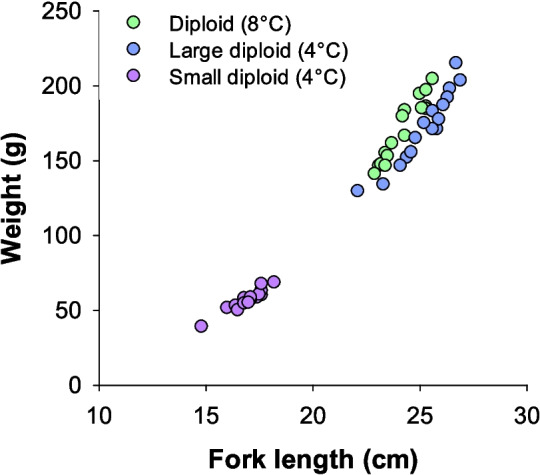
Weights versus fork lengths of individuals to illustrate the bimodal size distribution in the slow growing diploid group incubated at 4 °C. Approximately one-third of the group were smaller sized. These fish were sampled in August 2022 while the diploid group incubated at 8 °C was sampled in November 2021. *N* = 16

In the slow growing diploids incubated at 4 °C, a bimodal size distribution developed where approximately one-third of the group were notably smaller. Size parameters were measured in a subsample of 16 smaller fish in August 2022. Their weight was 57.0 ± 1.8 g, and their fork length was 17.0 ± 0.2 cm. In comparison, the larger-sized fish from the same tank that were tested with respirometry were weighing 172 ± 6 g and had a fork length of 25.2 ± 0.3 cm; thus, they were approximately three times as heavy and 50% longer than the smaller sized subgroup (Fig. 3).

### Metabolic rates and hypoxia tolerance

**Fig. 4:**
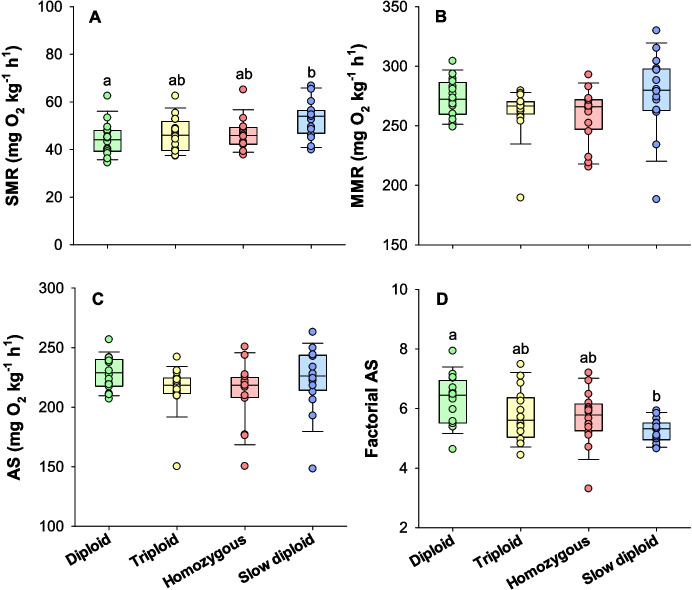
Metabolic rate traits of the isogenic Atlantic salmon groups. Standard metabolic rate (SMR) (**A**), maximum metabolic rate (MMR) (**B**), aerobic scope (AS) (**C**), and factorial AS (**D**). Different letters indicate statistical differences between groups (one-way ANOVA with Tukey test, *P* < 0.05). Figure panels show box plots together with individual data points (*N* = 16)

The standard metabolic rate was similar between the diploid, triploid, and homozygous groups that had been incubated at 8 °C, while the slow diploid group incubated at 4 °C had a significantly higher standard metabolic rate (52.6 ± 2.0 mg O_2_ kg^−1^ h^−1^) compared to the diploid group incubated at 8 °C (44.5 ± 1.8 mg O_2_ kg^−1^ h^−1^) (logged one-way ANOVA and Tukey test, *DF* = 61, *P* = 0.007) (Fig. 4A). The maximum metabolic rate during acute stress was not statistically different between all treatment groups (one-way ANOVA, *DF* = 63, *P* = 0.231) (Fig. 4B). The absolute aerobic scope was also statistically similar between all treatment groups (one-way ANOVA, *DF* = 63, *P* = 0.147) (Fig. 4C). However, the factorial aerobic scope was significantly lower in the slow diploids incubated at 4 °C (5.3 ± 0.1) when compared to diploids incubated at 8 °C (6.3 ± 0.2) (one-way ANOVA and Tukey test, *DF* = 63, *P* = 0.006) (Fig. 4D).

**Fig. 5:**
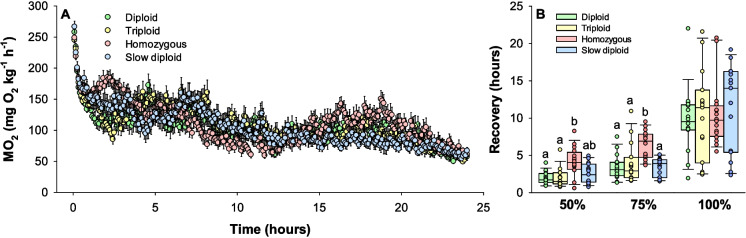
Oxygen uptake rates (ṀO_2_) over time in normoxia (**A**), and recovery time to baseline expressed as a percentage towards the standard metabolic rate (**B**). Data in **A** shows group mean ± standard error of the mean over time, and data in **B** are shown as boxplots together with individual data points. Statistical differences in **B** between groups within recovery thresholds are indicated with different letters (one-way ANOVA with Tukey test, *P* < 0.05) (*N* = 16)

An overview of the ṀO_2_ in normoxia in the first 24 h of the respirometry trials is shown in Fig. 5A. This period consisted of a substantially elevated ṀO_2_ at the beginning, highlighting the initial acute stress response, which then is followed by gradual recovery towards a resting baseline over several hours. To quantify the recovery trajectories between groups after acute stress, the time required to calm down (half, three-quarters, and fully) to the standard metabolic rate was calculated (Fig. 5B). The homozygous group (4.2 ± 0.5 h) was significantly slower to recover halfway towards the standard metabolic rate when compared to the diploids (2.0 ± 0.2 h) and triploids (2.0 ± 0.3) (one-way ANOVA and Tukey test, *DF* = 61, *P* = 0.004 and 0.02, respectively). Additionally, to recover three-quarters towards the standard metabolic rate took significantly longer in the homozygous group (6.4 ± 0.5 h) compared to all the other groups (3.6 ± 0.3 h) (one-way ANOVA and Tukey test, *DF* = 61, *P* < 0.005). However, time needed for full recovery was similar between all groups (one-way ANOVA, *DF* = 61, *P* = 0.835), being 10.5 ± 0.7 h across groups.

**Fig. 6:**
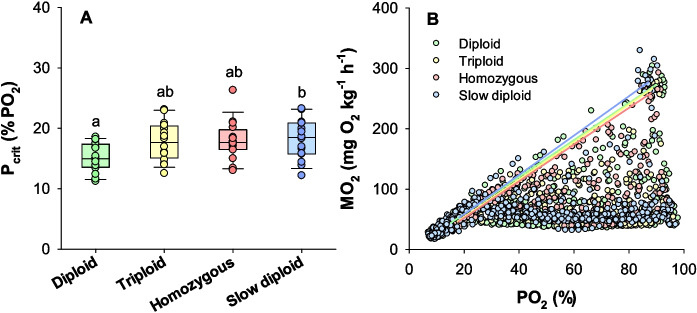
Response and tolerance to progressive hypoxia in the isogenic Atlantic salmon groups. Panel **A** shows the critical oxygen tension (*P*_crit_) as box plots together with individual data points. Significant differences are indicated with letters (one-way ANOVA with Tukey test, *P* < 0.05) (*N* = 16). Panel** B** shows scatter plots of all data points colored by clonal group along with lines between mean values of MMR and *P*_crit_

The *P*_crit_ was significantly higher in the slow diploids incubated at 4 °C (18.2 ± 0.8% O_2_) compared to diploids incubated at 8 °C (15.2 ± 0.6% O_2_) (one-way ANOVA and Tukey test, *DF* = 63, *P* = 0.030) while all other group comparisons were similar (*P* > 0.05) (Fig. 6A). A descriptive overview of the response to progressive hypoxia expressed as ṀO_2_ versus PO_2_ together with regression lines between the maximum metabolic rates in normoxia to the ṀO_2_ at *P*_crit_ is shown in Fig. 6B. Here, these regression lines indicate the highest attainable ṀO_2_ at various ambient oxygen levels, illustrating that the absolute aerobic scope decreases with decreasing oxygen levels towards zero at the *P*_crit_.

### Heart morphology

**Fig. 7:**
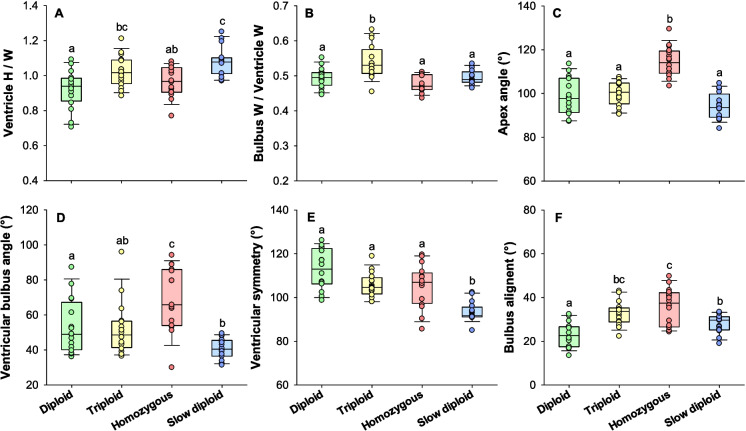
Heart morphology parameters of the isogenic Atlantic salmon groups. Panels show boxplots together with individual datapoints (*N* = 16). H = height and W = width. Statistical differences between groups are indicated with different letters (ANOVA with Tukey test, *P* < 0.05)

The ventricle height to width ratio was significantly higher in the slow diploids incubated at 4 °C compared to the diploid and homozygous groups incubated at 8 °C (one-way ANOVA and Tukey test, *DF* = 63, *P* < 0.006) (Fig. 7A), meaning that the latter groups had more rounded ventricles. The bulbus width to ventricle width ratio was significantly higher in the triploid group compared to the other groups (one-way ANOVA and Tukey test, *DF* = 63, *P* < 0.003) (Fig. 7B). Meanwhile, the apex angle (another measure of ventricular roundness) was significantly higher in the homozygous group compared to the other groups (one-way ANOVA and Tukey test, *DF* = 63, *P* < 0.001) (Fig. 7C).

The ventricular bulbus angle was significantly higher in the homozygous group compared to all the other groups (logged one-way ANOVA, *DF* = 62, *P* < 0.05), and moreover, the ventricular bulbus angle was significantly lower in the slow diploids incubated at 4 °C compared to the diploids incubated at 8 °C (*P* = 0.042) (Fig. 7D). Ventricular symmetry was significantly lower in the slow diploids compared to the other groups (ANOVA one ranks, *H* = 30,139, *DF* = 3, *P* < 0.001) (Fig. 7E). Furthermore, the bulbus alignment was significantly lower in the diploids incubated at 8 °C when compared to the other groups (logged one-way ANOVA and Tukey test, *DF* = 63, *P* < 0.023) (Fig. 7F).

As a final note, nearly all of the homozygous fish appeared to be extremely deviating when assessed qualitatively according to Engdal et al. (2024).

### Vaterite otolith deposition

**Fig. 8:**
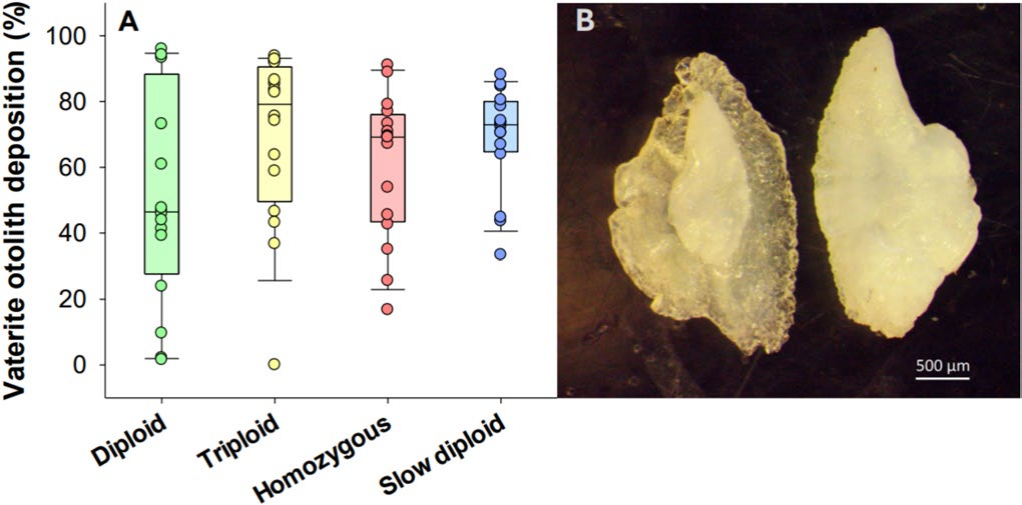
Panel **A** Percentage vaterite otolith deposition between isogenic groups shown as boxplots together with individual datapoints (*N* = 16). The treatment groups were not significantly different from each other (one-way ANOVA with Tukey test, *P* > 0.05). Panel **B** Representative photograph of an otolith showing vaterite (outer) and aragonite (central) depositions (left), and a healthy otolith only with aragonite (right)

There was no significant differences between any of the isogenic fish groups in the proportion of vaterite otolith deposition (one-way ANOVA, *DF* = 63, *P* = 0.131) (Fig. 8A). Furthermore, the two size groups incubated at 4 °C also did not differ (*t*-test, *t* = 1.578, *DF* = 30, *P* = 0.125), with vaterite proportions being 69.03 ± 3.93% in the larger group and 54.54 ± 5.01% in the smaller group. A representative photograph of a sagittal otolith with vaterite formation on the outer layer, and one with only aragonite, are shown in Fig. 8B. Overall, substantial variation in the proportion of vaterite between individual fish was found in all the isogenic groups, and only two out of 80 assessed fish had both sagittal otoliths with zero vaterite crystals.

### Individual variation in isogenic and outbred Atlantic salmon

**Fig. 9:**
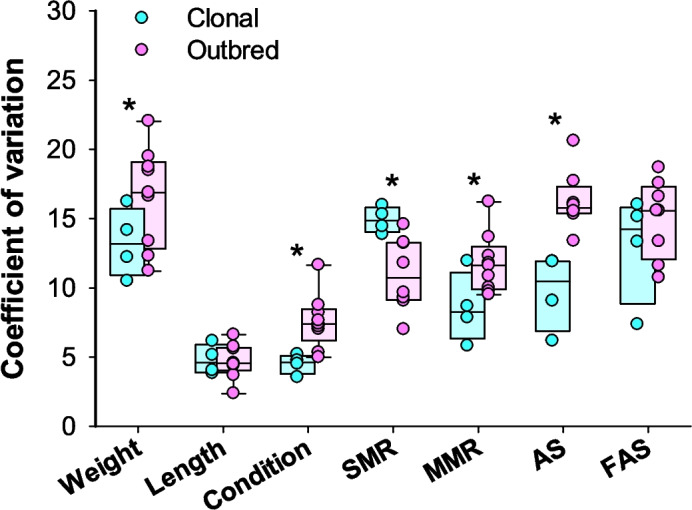
Coefficient of variation for size and metabolic rate parameters measured in the four isogenic Atlantic salmon groups (present study) and in outbred Atlantic salmon groups from other contemporary experiments using similar methods and protocols (Hvas and Bui 2022; Hvas et al. 2025). Statistical differences between clonal and outbred groups within a parameter is indicated with an asterisk (two-way ANOVA with Tukey test, *P* < 0.05). For all groups represented by a data point, *N* = 16

To explore whether the variation between individuals among isogenic fish was smaller than in conventionally outbred genotypes of Atlantic salmon, the coefficient of variation for size and metabolic rate parameters in the present study was compared with contemporary studies from our laboratory that used non-isogenic Atlantic salmon from a standard aquaculture stock (AquaGen) (Hvas and Bui 2022; Hvas et al. 2025) (Fig. 9). These experiments used the same methods and equipment for the parameters compared and also had an experimental design with *N* = 16 per treatment group as in the present study on isogenic fish. Across all parameters the coefficient of variation was significantly higher in outbred fish groups (two-way ANOVA, *DF* = 87, *P* < 0.001). Specific parameters that differed were weight, condition factor, maximum metabolic rate, and absolute aerobic scope that all had higher coefficients of variation in outbred groups than in isogenic groups (Tukey test, *P* = 0.026, 0.038, 0.032, < 0.001, respectively). On the contrary, the standard metabolic rate had a smaller coefficient of variation in outbred fish groups (*P* = 0.010). Meanwhile, length and factorial aerobic scope did not have significantly different coefficients of variation (Tukey test, *P* > 0.05).

## Discussion

### Metabolic phenotypes in isogenic Atlantic salmon

The isogenic fish groups were generally similar in the physiological traits measured. The most notable difference was that heterozygous diploids incubated at 4 °C had a higher standard metabolic rate than the heterozygous diploid group incubated at 8 °C. Owing to differences in standard metabolic rates, the derived *P*_crit_ and factorial aerobic scope were therefore also different between heterozygous diploids from the two incubation temperatures. However, both the *P*_crit_ and the factorial aerobic scope are known to be inherently sensitive to slight changes in standard metabolic rates, as opposed to the absolute aerobic scope (Halsey et al. 2018; Hvas 2022a). Caution is therefore warranted when interpreting physiological implications in cases were factorial and absolute aerobic scope provide opposing results between treatments (Clark et al. 2013; Halsey et al. 2018).

The difference in standard metabolic rates likely reflects differences in recent growth history prior to experimental trials. The 4 °C incubation group had been growing rapidly over the preceding summer months in higher temperatures following a long winter with little growth and were presumably still prioritizing substantial somatic growth based on fish being leaner with lower condition factors. The 8 °C group, on the other hand, at the time of testing had been experiencing gradually declining seasonal water temperatures in the autumn and had higher condition factors, making them prepared for overwintering where growth conditions are less optimal. Seasonal differences in standard metabolic rates in fish are known from earlier studies, and may vary with day-length, expected food availability, and spawning periods (Karaås 1990; Beamish 2011). In the present study, fish were maintained on continuous light following first-feeding meaning that the main difference between the two incubation treatments was the thermal growth history. Following recent prolonged exposure to ambient summer temperatures that are more optimal for growth, increases in anabolic processes could presumably then lead to higher basal metabolic costs, explaining a higher standard metabolic rate in the 4 °C incubation group. Furthermore, at the time of testing, fish in this group were approximately 9 months older than the other groups and may be considered to be in the process of “catching up” through compensatory growth mechanisms (Ali et al. 2003). During restricted feeding or prolonged fasting, for instance in winter, body length can continue to increase in Atlantic salmon resulting in leaner fish with lower condition factors which prepares them for rapid somatic growth once the environment allows it again (Einen et al. 1998; Hvas et al. 2022).

The triploid group was similar to the diploid heterozygous groups in all the metabolic traits measured. This was contrary to our hypothesis, as an extra set of chromosomes did not elevate basal maintenance costs nor did larger cells with a lower surface to volume ratio limit oxygen uptake capacities and hypoxia tolerance. Previous physiological studies on outbred triploid salmonids have often reported contrasting results. For instance, standard metabolic rates in triploid brook charr (*Salvelinus fontinalis*) have been reported higher than in diploids (O’Donnell et al. 2017), while another study found similar metabolic rates between triploids and diploids of this species (Hyndman et al. 2003). In Atlantic salmon, diploids and triploids have been reported to have similar metabolic rates and thermal tolerance (Bowden et al. 2018), while others found that triploids had lower maximum metabolic rates than diploids at 10.5 °C, but not at 3 °C (Riseth et al. 2020). Triploid Atlantic salmon have also been found to perform worse in hypoxia and elevated temperatures, and this was associated with overt ram ventilation suggesting they struggled with oxygen uptake (Hansen et al. 2015). These discrepancies could result from numerous factors in each study including random effects, genetic materials used, experimental methodologies, life stage, and rearing environment.

The homozygous diploid group had similar metabolic phenotypes as the heterozygous diploid and triploid groups except for slower stress recovery trajectories from acute stress exposure at intermediate time scales. Contrary to our hypothesis, being functionally completely inbred did therefore not result in obvious physiological impairment in the isogenic line tested here. We are unaware of previous reports on metabolic phenotypes in fully homozygous Atlantic salmon, but increased heterozygosity in various alleles has been linked with lower oxygen uptake rates and faster developmental growth in rainbow trout which was interpreted as increased metabolic efficiency in less inbred individuals (Danzmann et al. 1987). Curiously, similar patterns of lower oxygen consumption and improved growth in more heterozygous individuals has also been found in the coot clam (*Mulinia lateralis*) (Garton et al. 1984).

The initial slower stress recovery in homozygous Atlantic salmon may have some negative implications if they were subjected to repeat or multiple stressors within brief time periods. It should also be noted that the homozygous group had higher early-mortality rates while numerous individuals suffered obvious spinal deformities, and these individuals were avoided for the experimental trials. Some selection bias in choosing healthier individuals therefore occurred in the homozygous fish group, which may help explain why they performed better than anticipated in the respirometry trials.

### Heart morphology in isogenic Atlantic salmon groups

The heart morphology parameters assessed varied distinctly between the isogenic fish groups, pointing to that both zygosity, ploidy, and incubation temperatures differentially affected how the heart develops and functions in Atlantic salmon.

The slower developing heterozygous diploid group incubated at 4 °C had higher ventricle height to width ratios than the heterozygous diploid group incubated at 8 °C, signifying a more triangular shaped ventricle. This was expected, as a slower growth history previously has been shown to produce this phenotype while faster growing Atlantic salmon have more rounded ventricles (Brocklebank and Raverty 2002; Frisk et al. 2020; Vindas et al. 2024). Furthermore, the 4 °C incubation group had a lower ventricular symmetry and a lower ventricular bulbus angle than the other isogenic diploid groups, suggesting a strong environmental influence for these two morphology traits. A lower ventricular symmetry was previously observed in wild Atlantic salmon and in farmed fish subjected to slower growth conditions (Vindas et al. 2024; Hvas et al. 2025), while a lower ventricular bulbus angle also has been reported in wild Atlantic salmon (Poppe et al. 2003; Engdal et al. 2024). As such, slower developmental growth in the 4 °C incubation group resulted in heart morphology traits more resembling wild phenotypes.

We are unaware of previous assessments of heart morphology traits in homozygous salmonids. Here, nearly all fish in the homozygous group had extremely deviating heart shapes based on qualitative assessments according to Engdal et al. (2024). These deviations calls for some caution when interpreting the quantitative measurements of the heart shape. When measured qualitatively, the homozygous group had a higher apex angle and a higher ventricular bulbus angle than all the other groups, while the bulbus alignment also was higher than in the two heterozygous diploid groups.

The triploids differed from the other groups by having a higher bulbus width to ventricle width ratio. Previously, triploid Atlantic salmon were found to have a lower angle of the bulbus arteriosus and suffer increased risks of aplasia in the septum transversum at higher incubation temperatures than diploid counterparts (Fraser et al. 2013a, b).

The distinct heart morphology associated with being functionally inbred in the homozygous group or being more wildtype-like in the 4 °C incubation group was hypothesized to either worsen or improve the aerobic capacity, respectively, when measured in the respirometry trials. However, the maximum oxygen uptake rates during stress and the derived absolute aerobic scope were similar across the isogenic fish groups. Moreover, individual absolute aerobic scopes were not significantly correlated with any of the six heart morphology traits across all isogenic treatment groups.

It is presently unclear how distinct morphological phenotypes of the heart may influence the resultant physiological capacities at the whole-animal level in Atlantic salmon. However, triangular shaped ventricles are often found in active and migratory species of fish (Agnisola and Tota 1994; Sanchez-Quintana et al. 1995), and rainbow trout with more triangular shaped ventricles have been found to be better swimmers with higher oxygen uptake capacities and increased cardiac output compared to individuals with more rounded ventricles (Claireaux et al. 2005). Furthermore, heart morphology traits such as more rounded ventricles and increased ventricular symmetry is associated with higher mortality risks following stressful events in larger-sized farmed Atlantic salmon (Frisk et al. 2020).

Not finding a clear link between respiratory capacity and heart morphology traits could be caused by the experimental context of the present study. As such, certain phenotypes of the Atlantic salmon heart could cause a bottleneck in whole-animal performance traits in other scenarios, particularly at elevated less-optimal temperatures where oxygen demand becomes substantially higher (Hvas et al. 2017; Hvas 2022b), which then will assert greater strain on the cardiovascular system in situations of stress or high activity (Farrell 2002, 2007). Furthermore, considering that deviating heart morphology has been associated with increased mortality risk primarily in larger-sized farmed Atlantic salmon (Brocklebank and Raverty 2002; Frisk et al. 2020; Hvas et al. 2025), life stage and body size could be another important factor owing to physiological scaling effects that may modify which component of the integrated cardiorespiratory system that sets the limit of whole-animal performance traits. Owing to the distinct morphological phenotype of the heart in both homozygous and slower-developing isogenic Atlantic salmon found in the present study, it would be interesting in a future study to measure physiological performance on these fish groups at larger body sizes and at higher temperatures.

### Vaterite deposition in sagittal otoliths

Otolith deformities assessed by the proportion of vaterite deposition did not differ between isogenic fish groups while the individual variation was high across all groups. Only two out of 80 fish investigated did not have any vaterite deposition, meaning that the vast majority showed varying severities of deformed sagittal otoliths, as previously reported in farmed Atlantic salmon (Reimer et al. 2016). This may constitute concerns regarding fish welfare and performance, as abnormal otoliths are associated with hearing loss and impaired swimming balance (Tomas and Geffen 2003; Oxman et al. 2007; Brown et al. 2013).

Slower growth trajectories were previously found to reduce the proportion of the vaterite deposition in farmed Atlantic salmon (Reimer et al. 2017), suggesting that a slower and more natural early growth development will result in healthier phenotypes that more closely resemble wild fish. We therefore expected less vaterite in the fish incubated at 4 °C, but contrary to this hypothesis they did not differ from the fish groups incubated at 8 °C, nor were there any differences between small and big fish within the bimodal size-distribution.

The underlying cause for vaterite formation in fish otoliths is not fully understood. It has been suggested that it is related to imbalances in the ratio of scaffold proteins in the endolymph where otoliths develop (Reimer et al. 2017). During biomineralization of otoliths proper modification via phosphorylation of the *Starmaker-like* protein is imperative for aragonite becoming the crystal polymorph (Wojtas et al. 2012; Kalka et al. 2019; Thomas et al. 2019). However, for phosphorylation to correctly modify protein functioning, adequate amounts of adenosine triphosphate is required as substrate. Thus, accelerated growth conditions could limit the available substrate for protein phosphorylation, causing vaterite to become the dominant crystal polymorph. Conversely, it could also be that a highly repressed growth trajectory at the earliest life stages facilitated by low temperatures could impair adequate protein phosphorylation in otoliths, explaining why the proportion of vaterite also was high in fish incubated at 4 °C in the present study. To obtain optimal conditions for proper otolith development in Atlantic salmon, a more balanced intermediate growth trajectory might therefore be required. Another possibility is that certain cultured genotypes of Atlantic salmon are genetically predisposed to develop deformed otoliths, and that the isogenic fish lines used in the present study suffered this particular trait.

Considering that the data variation in vaterite proportions was remarkably high, more individuals would ideally have to be sampled in future studies to ensure statistical robustness. This defeats one of the expected advantages when using an isogenic animal model, which is a reduction in number of replicates (Festing 1999; Hubrecht and Carter 2019). Despite fish being genetically identical and maintained in a stable tank environment, random factors appeared to have played the biggest role in the phenotypic outcome of otolith morphology, similarly to how random factors previously caused high data variation in parasite infestation levels in both isogenic and outbred Atlantic salmon (Glover et al. 2017b).

### Emergence of a bimodal size-distribution in isogenic Atlantic salmon

The developmental rate of Atlantic salmon depends on the incubation temperature (Gorodilov 1996). Consequently, the heterozygous diploid Atlantic salmon incubated at 4 °C were first fed 3.5 months later than the isogenic fish groups incubated at 8 °C. Due to this shift in timing they also experienced a different temperature regime later in life. At the time of experimental trials, a clear bimodal size-distribution was present in the 4 °C group. In the wild, Atlantic salmon juveniles are known to exhibit bimodal size groupings owing to diverse life-history strategies in how long they spend in freshwater before migrating to sea (Thorpe 1977; Thorstad et al. 2011). This is dependent on body size in the autumn where smaller sized individuals may delay their seaward migration to another year (Metcalfe et al. 1988; Skilbrei 1991). Overwintering fish in freshwater can then go into prolonged periods of winter anorexia until spring, even when food is readily available (Metcalfe et al. 1992). As such, the smaller isogenic Atlantic salmon in the present study presumably fell below a certain threshold for nutritional status and body size as water temperature was declining in the autumn, signaling them to choose a different life-history strategy than conspecifics that were slightly larger during this critical period.

While salmon parr can be more aggressive and competitive over resources compared to later life stages (Turnbull et al. 1998; Cañon Jones et al. 2010; Hvas et al. 2024), fish here were fed ad libitum in a controlled tank environment with many individuals, and establishment of dominance hierarchies being the main cause for heterogenous growth therefore seemed unlikely. Moreover, owing to the fish being isogenic the emergence of a bimodal size distribution cannot be ascribed to a genetic component. Random effects leading to subtle variation in body sizes at a critical time point may instead have facilitated the large size disparity between the two groupings several months later.

### Individual variation in phenotypes and clonal fish lines as experimental models

Substantial individual variation within treatment groups is an inherent challenge that may obscure data interpretation in experimental biology (Festing 1976; 1999; Brown et al. 2011). Moreover, to account for variation and to ensure statistically robust data when designing experiments, a high number of replicates is preferred, but this goes against the 3Rs concept with regards to reduction in animal ethics (Hubrecht and Carter 2019). To reduce variation, isogenic fish lines should therefore provide an obvious advantage in many types of biological experiments (Komen and Thorgaard 2007; Franěk et al. 2020).

In the present study, we measured physiological and morphological traits of interest along with size parameters in isogenic Atlantic salmon groups. Owing to constant genetics as well as a stable laboratory rearing environment, we expected data to be highly consistent between individuals within each isogenic group, and that the coefficient of variation would be lower when compared to previous measurements on the same traits in outbred Atlantic salmon. However, despite having used isogenic fish in a stable laboratory rearing environment, an appreciable amount of data variation was still observed in all the traits measured. The amount of data variation also depended greatly on the phenotypic trait where vaterite deposits in otoliths showed a particularly high variation within groups.

When compared to outbred Atlantic salmon, the coefficient of variation was overall smaller across traits in the isogenic groups, as expected. Although this was not consistent for each specific trait. In previous studies, individual variation in body weight and length among isogenic fish groups has been found to be lower when compared to outbred fish group in Amago salmon (*Oncohynchus rhodurus*), Aya (*Plecoglossus altivelis*), and Nile tilapia (Kobayashi et al. 1994; Taniguchi et al. 1994; Muller-Belecke and Horstgen-Schwark 2000). However, higher individual variation has been reported for body weight and length in isogenic carps relative to outbred groups (Komen and Thorgaard 2007), as well as for meristic traits in isogenic rainbow trout relative to outbred groups (Young et al. 1995). In isogenic zebrafish, the variation in whole-body lactate concentrations following swim testing was similar to outbred zebrafish (Plaut and Gordan, 1994). It is therefore not given beforehand that isogenic fish will consistently show less individual variation than outbred counterparts.

It is interesting to consider that all the variation within group observed in the isogenic Atlantic salmon groups cannot be ascribed to a genetic component. Instead, this variation must stem from random factors in the environment together with potential inaccuracies in how the data was collected. Certain traits are evidently more influenced by random factors than others, and such seemingly random effects may play a deciding role in major phenotypic outcomes and overall fitness. For instance, in choice of life history as exemplified in the bimodally distributed body sizes in the heterozygous isogenic group incubated at 4 °C (present study), or in obtained parasite infestation levels (e.g., Glover et al. 2017b).

Regarding measurement inaccuracies and the role of human error in data collection, this issue would be expected to increase when measuring more complex traits with more difficult methodologies. However, even body weight, clearly one of the simplest traits to measure precisely, had a substantial coefficient of variation, and it was higher than, for instance, the coefficient of variation for maximum metabolic rate and absolute aerobic scope which both are more complicated to measure. Method accuracy is nevertheless important to consider, particularly when data variation is higher than anticipated and when genetic diversity cannot be blamed.

While some traits showed a lower coefficient of variation in the present study, the use of isogenic Atlantic salmon groups did not completely solve the prevailing challenge of high phenotypic variation within-groups in biological experiments. However, in our evaluation we still conclude that isogenic models can be useful, not least as any variation unrelated to a genetic component can provide novel insights of environmental effects on the phenotypic outcome.

## Data Availability

Data is available upon request to the corresponding author.
